# A Justified Protest

**Published:** 1908-07

**Authors:** 


### Correspondence.

### A Justified Protest.

SALTILLO, MEXICO, June 26, 1908.

*Editor Texas Medical Journal.*

SIR: The ability of the "Red Back"—that of "ye editor" as well, to answer, correctly, any and all questions put to it, coupled with a "longing" on my part, doubtless on the part of a large majority of the medical profession as well, compels me to inquire why is it that in "reviews" of medical publications the publishers, editors of medical journals, or the reviewers, one, or all of them, do not give the prices of the works reviewed?

As I understand it, these "reviews" are intended to inform the medical profession—the trade, if you please—of the merits or demerits of the books reviewed. If this is correct, then why not add another bit of useful information—why not "go the whole hog," and give the prices also?

In the *American Journal of the Medical Sciences* for April, May and June, 1908, twenty-seven medical books are reviewed, but the price is not given of a single one of them; and it so happened that I wished to add two of these books to my library, but in order to do so I was compelled to write to one publisher in Chicago and to another in Philadelphia to ascertain the prices before I could order them, whereas if the prices had been given in the "reviews," as they should have been, I could have put them into my library in one-half the time and with one-half the trouble to the respective publishers and to myself. I complained to these publishers of their foolish-want-of-business-methods in not having the prices of their wares included in the "reviews" of them, and they both replied that they invariably gave the prices of all books that they sent for review. The fault, then, of which I complain, would appear to be with the editor of the journals or with the reviewers. Be it where it may, it should be corrected.

The *British Medical Journal* contains a printed notice requesting publishers who send their publications for "review" to include the price, that it may be stated in the "reviews."

Now why can not our publishers, editors and reviewers establish a like rational-business-like-method, and thus save their patrons and readers unnecessary trouble and delay? I note, with great pleasure, that *it is the practice of the "Red Back" to give the prices of the books which it "reviews."* Selah.

Yours truly,

R. H. L. Bibb.

Answer—Negligence on the part of reviewers.—Daniel.

---

Alcoholism.—

℞ Pareldehydi, fl. ʒ vj.

Elix. aromatici, q. s. ad fl. ℥ iv.

M. Sig. Tablespoonful in water every hour or two.

Indications.—Used in mild delirium, restlessness and insomnia. —*Ex.*

